# Prompt apoptotic response to high glucose in SGLT-expressing renal cells

**DOI:** 10.1152/ajprenal.00615.2018

**Published:** 2019-03-13

**Authors:** Linnéa M. Nilsson, Liang Zhang, Alexander Bondar, Daniel Svensson, Annika Wernerson, Hjalmar Brismar, Lena Scott, Anita Aperia

**Affiliations:** ^1^Science for Life Laboratory, Department of Applied Physics, Royal Institute of Technology, Solna, Sweden; ^2^Science for Life Laboratory, Department of Women’s and Children’s Health, Karolinska Institutet, Solna, Sweden; ^3^Institute of Chemical Biology and Fundamental Medicine, Novosibirisk, Russia; ^4^Division of Renal Medicine, Department of Clinical Science, Intervention and Technology, Karolinska Institutet, Stockholm, Sweden

**Keywords:** apoptosis, hyperglycemia, podocytes, proximal tubular cells, sodium-dependent glucose transporter

## Abstract

It is generally believed that cells that are unable to downregulate glucose transport are particularly vulnerable to hyperglycemia. Yet, little is known about the relation between expression of glucose transporters and acute toxic effects of high glucose exposure. In the present ex vivo study of rat renal cells, we compared the apoptotic response to a moderate increase in glucose concentration. We studied cell types that commonly are targeted in diabetic kidney disease (DKD): proximal tubule cells, which express Na^+^-dependent glucose transporter (SGLT)2, mesangial cells, which express SGLT1, and podocytes, which lack SGLT and take up glucose via insulin-dependent glucose transporter 4. Proximal tubule cells and mesangial cells responded within 4–8 h of exposure to 15 mM glucose with translocation of the apoptotic protein Bax to mitochondria and an increased apoptotic index. SGLT downregulation and exposure to SGLT inhibitors abolished the apoptotic response. The onset of overt DKD generally coincides with the onset of albuminuria. Albumin had an additive effect on the apoptotic response. Ouabain, which interferes with the apoptotic onset, rescued from the apoptotic response. Insulin-supplemented podocytes remained resistant to 15 and 30 mM glucose for at least 24 h. Our study points to a previously unappreciated role of SGLT-dependent glucose uptake as a risk factor for diabetic complications and highlights the importance of therapeutic approaches that specifically target the different cell types in DKD.

## INTRODUCTION

Diabetic kidney disease (DKD) is the most common cause of chronic kidney disease and end-stage renal failure. It is associated with a large social and economic burden, and there is an unmet need for therapy to halt the progressive course of the disease (6, 37). DKD has been extensively studied during the last decade. However, there is no uniform concept regarding the cellular mechanisms behind the disease and its progressive course. The majority of studies have focused on the role of one cell type, omitting comparisons. However, given the complexity of the kidney, it is likely that there are several ongoing disease processes, and the development of a therapeutic program that prevents or halts the progressive course of DKD will need to be based on an insight into the ongoing disease processes in each of the target cells in the kidney.

Podocytes, proximal tubule cells (PTCs), and mesangial cells (MCs) are the most commonly studied cells in DKD. Damage and loss of podocytes cause proteinuria and contribute to glomerulosclerosis (23, 47). Damage of tubular cells causes interstitial fibrosis and glomerular tubular dissociation (7, 34). Damage of MCs leads to mesangial expansion and contributes to glomerulosclerosis (1, 31). Hyperglycemia and insulin resistance are the main causes of diabetic complications (8, 41, 43). Tight glucose control reduces the overall incidence of micro- or macroalbuminuria and halts the progression to end-stage disease (38). Several factors mediate glycemic toxicity, including metabolic dysregulation and generation of advanced glycosylation end products (9). The question of whether the adverse effects of glucose concentrations, exceeding the levels in individuals who are nondiabetic, will also depend on the cellular mechanisms for glucose uptake has often been discussed but has rarely been addressed experimentally. PTCs, which have a high level of aerobic metabolism because of high reabsorption workload (19, 27), take up glucose via Na^+^-dependent glucose transporters (SGLTs) (24). MCs have also been reported to express SGLTs (20). Podocyte glucose uptake occurs via insulin-sensitive glucose transporter (GLUT)4 (14).

Glucose-related apoptosis was first reported in 1997 by Ortiz and Neilson (36), who showed that immortalized murine renal epithelial cells exposed to 25 mM glucose for at least 24 h caused upregulation of the apoptotic protein Bax, downregulation of the antiapoptotic protein Bcl-x_L_, and triggered apoptosis. Subsequently, most studies of renal apoptosis in DKD have been performed on immortalized renal cells exposed to glucose concentrations that generally by far exceed those commonly observed in the clinical setting. Here, we describe the early response of PTCs, MCs, and podocytes exposed to moderately high (10 and 15 mM) glucose concentrations. All experiments were carried out on primary cells, because cell lines undergo mutations, progressively lose their phenotype, and have a shift to more anaerobic metabolism. The onset of the mitochondrial apoptotic pathway was used to validate the response to high glucose, because apoptosis marks the transition from reversible to irreversible cell damage and is a common finding in studies of rodent models of DKD (7, 21).

## MATERIAL AND METHODS

### 

#### Antibodies and chemicals.

The following primary antibodies and dilutions were used: mouse monoclonal anti-Bax (6A7) (5 μg/ml), rabbit polyclonal anti-Bax (1:100), rabbit polyclonal anti-SGLT1 (1:50), rabbit polyclonal anti-SGLT2 (1:100), rabbit polyclonal anti-GLUT4 (1:500), and mouse monoclonal anti-α-smooth muscle actin (1:100) (all from Abcam, Cambridge, UK), rabbit polyclonal anti-SGLT2 (1:100, Fitzgerald Industries, Acton, MA), rabbit monoclonal anti-Bcl-x_L_ (54H6) (1:200, Cell Signaling Technology, Danvers, MA), rabbit polyclonal anti-Wilms tumor 1 (WT1; 1:200, Santa Cruz Biotechnology, Santa Cruz, CA), sheep polyclonal anti-nephrin (1:200, R&D Systems, Minneapolis, MN), and rabbit polyclonal anti-synaptopodin (1:500; Sigma-Aldrich, St. Louis, MO). The following fluorescence secondary antibodies were used: Alexa Fluor 488 goat anti-rabbit IgG, Alexa Fluor 546 goat anti-mouse IgG, and Alexa Fluor 546 goat anti-rabbit IgG (all from Life Technologies, Carlsbad, CA), Star 635P goat anti-rabbit IgG (Abberior, Göttingen, Germany), and Star 635P conjugated to donkey anti-sheep IgG (ThermoFisher Scientific, Waltham, MA) (all used at a concentration of 1:500). All antibodies used are commercially available and were validated by each manufacturer.

All chemicals and reagents were purchased from Sigma-Aldrich, and all cell culture and molecular biology materials were purchased from ThermoFisher Scientific, unless otherwise stated.

#### Microscopy.

A Zeiss LSM 510 confocal microscope equipped with ×25/0.8 numerical aperture (NA) oil, ×40/1.3 NA oil, ×63/1.4 NA oil, and ×40/1.2 NA water objectives was used for all imaging of PTCs, MCs, primary podocytes, and patient material. A Zeiss LSM 780 confocal microscope equipped with ×20/0.8 NA air and ×40/1.2 NA water objectives was used for all imaging of podocyte cell line. Immunofluorescence was detected as follows: cyan fluorescent protein (CFP) with excitation at 405 nm and detection at 454–580 nm, DAPI and NucBlue with excitation at 405 nm and detection at 420–480 nm, Alexa Fluor 488 with excitation at 488 nm and detection at 510–550 nm, Alexa Fluor 546 and TUNEL labeling with excitation at 543 nm and 575-nm long-pass detection, and Star 635P and DRAQ5 with excitation at 633 nm and 650-nm long-pass detection. JC-1 fluorescence ratios were recorded with excitation at 488 nm and simultaneous 505- to 530-nm and 560-nm long-pass detection. 2-[*N*-(7-nitrobenz-2-oxa-1,3-diazol-4-yl)amino]-2-deoxyglucose (2-NBDG) fluorescence was detected with 488-nm excitation and 505-nm long-pass detection, and di(acetoxymethyl ester) 6-carboxy-2′,7′-dichlorodihydrofluorescein diacetate (DCFDA) fluorescence was recorded with 488-nm excitation and 505- to 550-nm detection.

#### Animals and primary cultures.

Twenty-day-old male Sprague-Dawley rats were used for primary cell preparations. All animals were housed under controlled conditions of light and dark (12:12 h) and given a standard diet containing 20% protein by weight, and tap water was available ad libitum. All experiments were performed according to Karolinska Institutet regulations concerning the care and use of laboratory animals and were approved by the Stockholm North ethical evaluation board for animal research.

Primary culture of rat PTCs were prepared as previously described (11). PTCs were characterized after 3 days in culture; 99% of cells were SGLT2 positive (11).

Glomeruli isolation and podocyte culture were performed as follows: rats were anesthetized by intraperitoneal injection of pentobarbital and perfused through the left ventricle with HBSS to clear out blood followed by a solution of HBSS containing Dynabeads M-450. For each animal, 8 × 10^7^ Dynabeads in 20 ml of solution were used. After perfusion, the kidneys were removed, and the medulla was discarded. The cortex was cut to small pieces and digested in 1 mg/ml collagenase type I and 10 U/ml DNase in HBSS at 37°C for 30 min with gentle shaking. The digested tissue was gently pressed through a 100-μm cell strainer (BD Falcon, Bedford, MA). Glomeruli containing Dynabeads were collected using a magnetic particle concentrator, washed three times with cold HBSS, and seeded on 12- or 18-mm glass coverslips in 12- or 24-well petri dishes. Podocytes migrated out of glomeruli and were cultured for 3 days in pH 7.4 MEM-NEEA supplemented with 3.6 g/l HEPES, 0.5% insulin-transferrin-selenium-sodium pyruvate, 0.5% sodium pyruvate, 5% FBS, 10 µg/ml penicillin, and 10 µg/ml streptomycin in 37°C at an approximate humidity of 95–98% with 5% CO_2_.

Primary MC cultures were prepared from isolated glomeruli. Glomeruli were decapsulated by mixing the glomerular suspension with a 1-ml syringe and a 21-gauge needle a couple of times, resuspended in HBSS containing 1 mg/ml collagenase type I, and digested at 37°C for 15 min with gentle shaking. Cells were resuspended in DMEM supplemented with 2 mM l-glutamine, 20% FBS, 10 µg/ml penicillin, and 10 µg/ml streptomycin and plated in six-well plates. Cells were cultured in 37°C at an approximate humidity of 95–98% with 5% CO_2_, and culture media were changed every 48 h. After 7 days in culture, each well of cells was split (1:3) in the following way. Cells were washed with Ca^2+^- and Mg^2+^-free PBS (pH 7.4) and incubated in 1 ml/well Ca^2+^- and Mg^2+^-free PBS containing 0.05% trypsin and 0.02% EDTA for 1 min at 37°C. Most of the trypsin solution was removed, and cells were incubated for another 3 min. Culture medium containing FBS was added to stop the digestion, wells were split, and new culture medium was added. On the third passage, cells were seeded on 12- or 18-mm glass coverslips in 12 or 24-well plates for experiments. Cells were characterized using SGLT1 and α-smooth muscle actin antibodies, indicating that they were MCs.

In all experiments using PTCs or podocyte cultures, treatment was started on *day 2* or *3* in culture. MC cultures were used after being passaged three times. Cells were incubated using the following concentrations: 10–30 mM d-glucose and/or 2.5 mg/ml delipidated endotoxin-free albumin (Sigma-Aldrich) with or without 5 nM ouabain (Sigma-Aldrich), 1 µM dapagliflozin (Selleckchem, Munich, Germany), or 0.2 mM phlorizin (Selleckchem, Munich, Germany) for 2–24 h, as indicated in each figure. As controls, 5.6 mM glucose with or without 9.4 mM mannitol was used. Dapagliflozin and phlorizin were dissolved in DMSO, and an equal amount DMSO was added to all samples in those experiments as a control. Cultures were randomly divided between treatment groups for each experiment.

#### Immortalized murine podocytes.

We use a well-described and characterized immortalized mouse podocyte cell line (33). Cells were maintained and differentiated as previously described (26) with the following modifications. The culture medium was glucose-free RPMI-1640 supplemented with 5.5 mM d-glucose, 10% FBS, 10 µg/ml penicillin, 10 µg/ml streptomycin. For undifferentiated cells, 10 U/ml interferon-γ (Sigma-Aldrich) was used. Cells were differentiated for 7–14 days. Differentiated immortalized podocytes were transiently transfected with SGLT2-ires-CFP (GenScript, Piscataway, NJ) or empty vector CFP (Addgene, Cambridge, MA). DNA plasmids were delivered to the cells using Lipofectamine LTX reagent with plus reagent (ThermoFisher) diluted in Opti-MEM (ThermoFisher) according to the manufacturer’s instructions. The final DNA concentration in each well was 500 ng/ml. Cells were transfected for 48 h and characterized with SGLT2-ires-CFP fluorescence and anti-SGLT2 antibodies.

#### Immunocytochemical staining.

After treatment, cells were fixed with 4% paraformaldehyde (pH 7.4) and washed three times with PBS. Cells were permeabilized with 0.3% Triton X-100 for 10 min, washed three times, and blocked with 5% BSA in 0.1% Triton X-100 for 1 h. Primary antibodies were applied overnight at 4°C. Cells were washed three times, and secondary antibodies were applied for 1 h at room temperature. Secondary antibody controls were subjected to the same treatment, but primary antibodies were omitted. Cells were washed three times, mounted with Immu-Mount (Thermo Shandon, Midland, ON, Canada), and imaged with a confocal microscope. In some experiments, cells were counterstained with 1 µg/ml DAPI (Santa Cruz Biotechnology) for 1–2 min before being mounted.

#### Glucose uptake.

Cells were incubated with 100 µM 2-NBDG (Life Technologies) in Na^+^ buffer (135 mM NaCl, 5 mM KCl, 1 mM MgSO_4_, 0.4 mM K_2_HPO_4_, 5.5 mM glucose, 20 mM HEPES, and 1 mM CaCl_2_) or Na^+^-free buffer (NaCl changed for 135 mM choline chloride) (pH 7.4) for 1 h at 37°C. During the last 30 min of incubation, 2 drops/ml of NucBlue Live ReadyProbes Reagent (NucBlue, Life Technologies) were added to the buffer for nuclear stain. Cells were washed once with Na^+^ or Na^+^-free buffer and imaged with a confocal microscope with fixed settings for all measurements. Glucose uptake was quantified as mean fluorescent intensity of all cells in five to six separate areas on each coverslip and expressed as follows: Na^+^-dependent glucose uptake = [1 – (2-NBDG fluorescence in the absence of Na^+^/2-NBDG fluorescence in the presence of Na^+^)] × 100%. The average number of cells analyzed from each coverslip was 24 for PTCs, 10 for MCs, and 17 for podocytes.

#### Detection of apoptotic cells in culture.

Cells were fixed in methanol (Solveco, Rosersberg, Sweden) for 5 min at 4°C and in ethanol-acetic acid (2:1, Solveco) for 5 min at −20°C. After each fixation step, cells were washed with PBS a couple of times. The apoptotic index (AI) was determined with an ApopTag Red In Situ Apoptosis Detection kit (TUNEL, Merk Millipore, Billerica, MA) according to the manufacturer’s instructions. Cells were counterstained with 1 µg/ml DAPI for 1–2 min, mounted with Immu-Mount, and imaged with a confocal microscope. Cells were considered apoptotic when they expressed TUNEL staining and characteristic apoptotic morphology with condensed nuclei. The total number of cells was determined by DAPI staining, and the AI was calculated as the percentage of apoptotic cells. For each coverslip, 3–5 separate areas with at least 100 cells in each image were analyzed except for primary podocytes, where ~40–50 cells in each image were analyzed. To determine the AI of podocytes, podocytes were identified by immunostaining for WT1. Only podocytes outside of a glomerulus and positive for WT1 were included in AI calculations, because the total number of podocytes located inside a glomerulus is not possible to determine in this preparation.

#### SGLT2 knockdown in PTCs.

SGLT2 gene expression was transiently suppressed using a cocktail of designated siRNAs (Stealth siRNA, catalog nos. RSS329982, RSS329983, and RSS329984, ThermoFisher). The constructs were delivered into the cells using Lipofectamine RNAiMAX transfection reagent (ThermoFisher) diluted in Opti-MEM (ThermoFisher) according to the manufacturer’s instructions. Briefly, the transfection mixture was added to the cell culture medium (10% FBS) at final concentrations of 30 nM for each siRNA. Control cells were transfected with 90 nM of a nontargeting construct (Stealth RNAi siRNA Negative Control, Med GC, ThermoFisher). Cells were transfected for 48 h before glucose treatment.

#### Bax and Bcl-x_L_ abundance and translocation assessment.

PTCs were incubated with mitochondrion-targeted green fluorescent protein CellLight Mitochondria-GFP BacMam (Life Technologies, Grand Island, NY) overnight at 37°C on *day 2* in vitro and treated with glucose and/or albumin on *day 3* in vitro. MCs were incubated with BacMam after being seeded on coverslips for 3–4 days and incubated with glucose the following day. At the end of treatment, cells were fixed and immunostained for Bax or Bcl-x_L_. The microscope setting was kept fixed for all measurements. Bax translocation to the mitochondria was analyzed with Matlab (The MathWorks, Natick, MA) and calculated as the percentage of overlapping Bax (pixels) with mitochondria (pixels) normalized to cell size (pixels). The total abundance of Bax and Bcl-x_L_ was calculated as the percentage of Bax or Bcl-x_L_ (pixels) normalized to cell size (pixels). On each coverslip, at least three cells were analyzed. The control group was set to 100%.

#### Mitochondrial membrane potential detection.

The maintenance of mitochondrial membrane potential was determined with JC-1 dye (Lifetime Technologies). JC-1 dye is a cationic carbocyanine dye that accumulates to the mitochondria. At low concentrations, the dye is monomeric, causing green (527 nm) fluorescence. At high concentration, the dye aggregates, causing a red fluorescence emission (590 nm). Depolarization of the mitochondrial membrane is observed as a decrease in the red-to-green fluorescence ratio. After glucose treatment, cells were washed with Krebs-Ringer (pH 7.4) and incubated in culture medium containing 2.5 µg/ml JC-1 for 15 min at 37°C. Cells were subjected to live cell imaging using a confocal microscope with fixed settings. The mitochondrial membrane potential change was quantified as the red (polarized)-to-green (depolarized) intensity ratio using Matlab. For each coverslip, six separate areas were analyzed. All groups were normalized to control.

#### Detection of reactive oxygen species.

Reactive oxygen species (ROS) were measured with DCFDA (ThermoFisher Scientific), where intracellular ROS causes nonfluorescent DCFDA molecules to convert to a green fluorescent form. After glucose treatment, cells were incubated with 10 µM DCFDA and counterstained with 2 drops/ml NucBlue for 30 min at 37°C. Cells were rinsed twice with PBS before being subjected to live cell imaging using a confocal microscope with fixed settings for all measurements. ROS were quantified as the mean DCFDA intensity in each image. For each coverslip, at least eight individual areas were analyzed. All groups were normalized to control.

#### PCR.

Cells and tissue samples were collected, and mRNA was extracted and purified with the RNeasy mini kit (Qiagen, Hilden, Germany) following the manufacturer’s instructions. PTCs were collected as a positive control for SGLT2, rat intestine tissue was collected as a positive control for SGLT1, and Cos7 were collected as a negative control for SGLT1 and SGLT2. Reverse transcription was performed using an Iscript cDNA synthesis kit (Bio-Rad Laboratories, Solna, Sweden) following the manufacturer’s instructions using 1 µg sample as the starting material. The PCR mix was as follows: 1× Phusion GC buffer, 0.2 mM dNTPs, 1.2 mM MgCl_2_, 0.5 µM each for forward and reverse primer, 5% glycerol, 0.02 U/µl Phusion polymerase, 2 µl reverse transcription product for each 50-µl preparation, and sterile water. Glycerol was used to prevent aggregation of primers, PCR template, and PCR product.

Great care was taken to identify specific primers that yielded only one possible PCR product according to Primer Blast. The following primers were used: SGLT1, forward 5′-AGTCTACGTAACAGCACAGAAGAGC-3′ and reverse 5′-CTTCCTCCTCTTCCTTAGTCATCTT-3′; and SGLT2, forward 5′-CTCTAACATCGCCTACCCACG-3′ and reverse 5′-AGAAAGCACCCTTCTCATTAACAC-3′.

The PCR program was as follows: 98°C for 30-s hot start, 35 cycles at 98°C for 10 s, 63°C for 15 s, and 72°C for 30 s, finally 72°C for 3 min with a 4°C hold.

PCR products were separated on an agarose gel and visualized using SYBR green I nucleic acid gel stain. The expected PCR product for SGLT1 is 199 bp and for SGLT2 is 377 bp. PCR products were purified using a GeneJET PCR purification kit following the manufacturer’s instructions, sequenced (KI-gene, Solna, Sweden), and matched with the expected product (Nucleotide Blast) for verification.

#### Quantitative real-time RT-PCR.

Cells were transfected with SGLT2 siRNA (catalog no. RSS329983, ThermoFisher) or nontargeting construct for 48 h and collected, and mRNA were extracted and purified with the RNeasy mini kit following the manufacturer’s instructions. RNA concentration was determined using the Qubit RNA HS assay kit and Qubit 3.0 fluorometer. Samples were subjected to one-step quantitative real-time RT-PCR measurements using a Quant-X One Step qRT-PCR SYBR Kit (Clontech Laboratories, Mountain View, CA) on a C1000 Touch Thermal Cycler (Bio-Rad). Each sample was analyzed in duplicate, and SGLT2 expression was analyzed using the ΔΔC_t_ method (where C_t_ is threshold cycle) previously described by Pfaffl (39) with GAPDH as the housekeeping gene. The following primers were used for SGLT2: forward 5′-CTCTAACATCGCCTACCCACG-3′ and reverse 5′-AGAAAGCACCCTTCTCATTAACAC-3′; Rn_Gapd_1_SG QuantiTect Primer Assay (Qiagen, Hilden, Germany) was used for the GAPDH.

#### Statistical analysis.

All data are expressed as means ± SE. Significance was determined with one-way ANOVA (single treatment) or two-way ANOVA (multiple treatments) followed by a *t*-test when applicable. In experiments with only two sample groups, a *t*-test was used. Statistical significance levels were *P* < 0.05, *P* < 0.01, or *P* < 0.001 as indicated.

## RESULTS

**Fig. 1.**
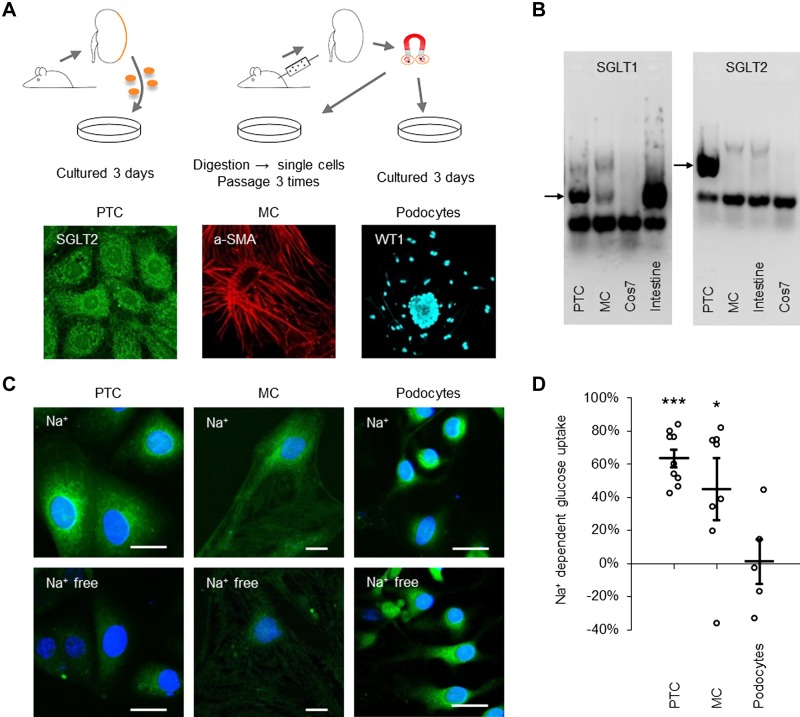
Cell preparation and documentation of Na^+^-dependent glucose transporter (SGLT) expression in proximal tubule cells (PTCs) and mesangial cells (MCs). *A*: PTCs (*left*) were prepared by digesting the outer cortex (150 µm) of rat kidneys into single cells, with cells allowed to culture for 2–3 days before being characterized with SGLT2 antibodies. MCs (*middle*) were prepared by perfusing rats with magnetic beads, extracting glomeruli-containing beads with a magnetic collector, and digesting glomeruli to single cells. Next, *passage 3* MCs were characterized with α-smooth muscle actin (α-SMA) antibodies. Podocytes (*right*) were prepared from extracted glomeruli as for MCs. Glomeruli were plated for 3 days, letting podocytes move out from the glomerulus. Podocytes were characterized with Wilms tumor 1 (WT1) antibodies. *B*: PCR for SGLT1 (*left*) and SGLT2 (*right*) in PTCs, MCs, Cos7, and intestine tissue, as indicated. Arrows show bands at 199 bp for SGLT1 and 377 bp for SGLT2. *C*: glucose uptake in PTCs (*left*), MCs (*middle*), and podocytes (*right*) measured with 2-[*N*-(7-nitrobenz-2-oxa-1,3-diazol-4-yl)amino]-2-deoxyglucose (green) in Na^+^ (*top*) or Na^+^-free (*bottom*) buffer (5.6 mM glucose). Cells were counterstained with NucBlue (blue). Scale bars = 20 µm. *D*: quantification of Na^+^-dependent glucose uptake in PTCs, MCs, and podocytes. Data are expressed as means ± SE; *n* = 9 coverslips for PTCs and *n* = 8 coverslips for MCs from 3 individual cell preparations and *n* = 5 coverslips from 2 individual cell preparations for podocytes. *P < 0.05; ***P < 0.001.

Our study was performed on primary cells from rat kidneys. The preparation of the cells is shown in Fig. 1*A*. PTCs and podocytes were studied within 3 days after plating and MCs were studied in *passage 3*, after other glomerular cells had been eliminated. All cells were cultured in medium containing 5.6 mM glucose before the study. Primary PTC cultures were prepared from the outer 150 µm of the renal cortex, which has ~90% proximal tubule volume (3) and generates a culture where 99% of the cells express SGLT2 during the first 3 days after plating (11). The late proximal tubular segments express SGLT1. MCs have been reported to express either SGLT1 or SGLT2 (44, 45). PTCs cultured for 3 days stained for SGLT2 and MCs stained for SGLT1 (Supplemental Figure S1, *A* and *B*; all Supplemental Material is available online at http://doi.org/10.6084/m9.figshare.7498271). PCR demonstrated the presence of SGLT1 and SGLT2 mRNA in the PTC sample and SGLT1 but not SGLT2 mRNA in the MC sample (Fig. 1*B*). PCR products were sequenced for verification. To verify that cells expressed functional SGLTs, basal glucose uptake was determined in all cell types in the presence and absence of Na^+^. Experiments were performed in triplicate and repeated three times. Approximately 60% of PTC glucose uptake and ~40% of MC glucose uptake was Na^+^ dependent; podocyte glucose uptake was Na^+^ independent (Fig. 1, *C* and *D*).

**Fig. 2.**
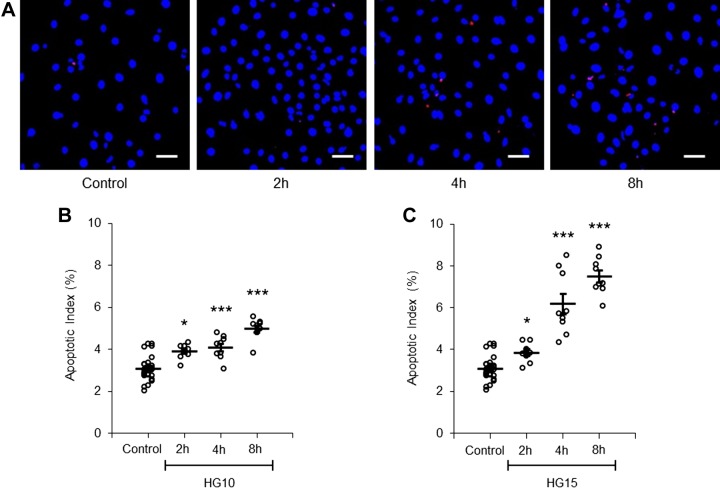
Short-time apoptotic response of proximal tubule cells (PTCs) to increased glucose concentration. *A*: PTCs were stained with TUNEL (red) and DAPI (blue). PTCs were incubated with control (5.6 mM) or 15 mM glucose-containing medium for 2, 4, and 8 h. Scale bars = 40 µm. *B* and *C*: quantification of the apoptotic index in PTCs incubated with control or with 10 mM (*B*; HG10) or 15 mM (*C*; HG15) glucose-containing medium for 2, 4, and 8 h. Approximately 100–200 cells in 5 separate areas of each coverslip were counted. Data are expressed as means ± SE; *n* = 9 coverslips from 3 individual cell preparations. *P < 0.05; ***P < 0.001.

**Fig. 3.**
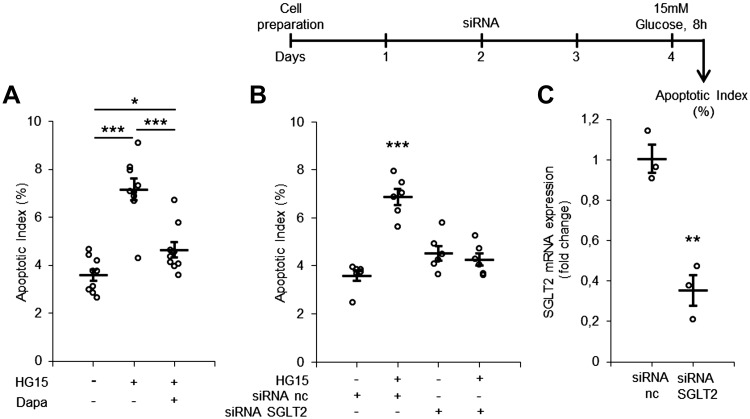
Na^+^-dependent glucose transporter (SGLT2) inhibition with dapagliflozin or knockdown with siRNA protects from high glucose-induced apoptosis in proximal tubule cell (PTCs). *A*: quantification of the apoptotic index in PTCs incubated with control (5.6 mM), 15 mM glucose (HG15)-containing, or 15 mM glucose + 1 µM dapagliflozin (Dapa)-containing medium for 8 h. Dapagliflozin was dissolved in DMSO; an equal amount DMSO was added to all samples as a control. Data are expressed as means ± SE; *n* = 9 coverslips from 3 individual cell preparations. *B*: timeline for siRNA silencing (*top*) and quantification of the apoptotic index in PTCs transfected with SGLT2 or negative control (nc) siRNA for 48 h and incubated with control or 15 mM glucose for 8 h (*bottom*). Data are expressed as mean ± SE; *n* = 6 coverslips from 2 individual cell preparations. *C*: quantification of SGLT2 mRNA expression after siRNA exposure for 48 h. Data are expressed as means ± SE; *n* = 3 cell preparations. **P < 0.01; ***P < 0.001.

We first tested the apoptotic effect of short-term exposure to a moderately increased glucose concentration (10–15 mM) in PTCs. Cells were TUNEL stained for the determination of the AI. A time- and concentration-dependent increase in the AI was recorded (Fig. 2 and Supplemental Figure S2). Because PTCs can take up glucose via SGLTs as well as GLUTs, we questioned whether preventing SGLT-mediated glucose uptake could protect from apoptosis. The apoptotic effect of high glucose exposure to increased extracellular glucose concentration was almost completely abolished in PTCs coincubated with glucose and the SGLT2 inhibitor dapagliflozin compared with glucose alone (Fig. 3*A*). To further validate the role of SGLT2 in glucose-triggered apoptosis, we downregulated SGLT2 expression in PTCs with siRNA for 48 h. siRNA treatment reduced SGLT2 mRNA levels (Fig. 3*C*) and prevented glucose-induced apoptosis (Fig. 3*B*). The level of the AI in control cells with SGLT2 siRNA and negative control treatment was comparable. The possibility that increased osmotic pressure was responsible for the apoptotic effect was excluded by parallel experiments using mannitol instead of glucose (Supplemental Table S1). Because few studies have documented the relevance of apoptosis in human DKD, we reexamined biopsy specimens from five male patients with DKD with regard to apoptosis and compared them with biopsies from three male healthy kidney donors at a corresponding age (Supplemental Figure S3*B*). The number of tubules with apoptotic cells in patients with DKD was threefold higher than in control individuals (Supplemental Figure S3, *A* and *C*). An aggregation of apoptotic cells in cross-sections of the tubular lumen was often observed in biopsies from patients with DKD (Supplemental Figure S3*D*).

**Fig. 4.**
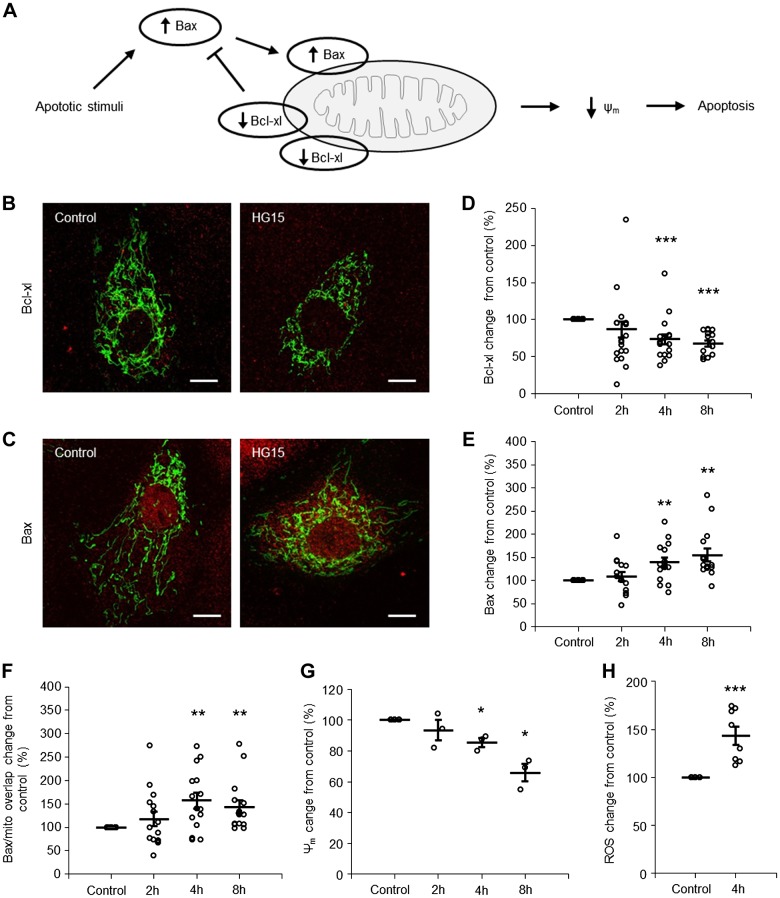
High glucose triggers apoptosis via the mitochondrial pathway in a time-dependent manner in proximal tubule cell (PTCs). *A*: cartoon illustrating activation of the mitochondrial apoptotic pathway. Under normal conditions, there is a balance between Bcl-x_L_ and Bax, preventing apoptosis. When an apoptotic stimulus, i.e., high glucose, activates the intrinsic apoptotic pathway, the balance between Bax and Bcl-x_L_ is disrupted, which leads to mitochondrial dysfunction [decreased mitochondrial membrane potential (Δψ_m_)] and apoptosis. *B* and *C*: immunofluorescence staining for Bcl-x_L_ (*B*) and Bax (*C*) expression (red) in PTCs incubated with control (5.6 mM) or 15 mM glucose (HG15)-containing medium for 8 h. Mitochondria are shown in green. Scale bars = 10 µm. *D−F*: quantification of Bcl-x_L_ abundance (*D*), Bax abundance (*E*), and Bax accumulation on mitochondria (*F*) in PTCs incubated with control or 15 mM glucose-containing medium for 2, 4, and 8 h. Data are expressed as means ± SE; *n* = 15 coverslips from 5 individual cell preparations. *G*: quantification of Δψ_m_ in PTCs incubated with control or 15 mM glucose-containing medium for 2, 4, and 8 h. Data are expressed as means ± SE; *n* = 3 coverslips from 3 individual cell preparations. *H*: quantification of ROS in PTCs incubated with control or 15 mM glucose-containing medium for 4 h. Data are expressed as means ± SE; *n* = 8 coverslips from 2 individual cell preparations. *P < 0.05; **P < 0.01; ***P < 0.001.

Several lines of evidence suggest that hyperglycemic toxicity is associated with activation of the mitochondrial apoptotic pathway controlled by the Bcl family of proteins, to which the apoptotic protein Bax and antiapoptotic protein Bcl-x_L_ belong (12, 16). Under healthy conditions, Bcl-x_L_ mainly resides on the mitochondria, whereas Bax is located both in the cytosol and on mitochondria. During the course of apoptosis, the abundance of Bcl-x_L_ decreases and abundance of Bax increases, allowing Bax to translocate from the cytosol to the mitochondria, where it will ultimately permeabilize the mitochondrial membrane, which marks the point of no return in the apoptotic process (Fig. 4*A*). Figure 4, *B* and *C*, shows PTCs immune stained for Bcl-x_L_ and Bax, respectively. Mitochondria were visualized with mitochondrion-targeted green fluorescent protein. Quantification of the fluorescent signals showed decreased expression of Bcl-x_L_ and increased expression and mitochondrial location of Bax in PTCs after 4–8 h of exposure to 15 mM glucose (Fig. 4, *D–F*). The ongoing apoptotic process was accompanied by a decrease in mitochondrial membrane potential and an increase of ROS (Fig. 4, *G* and *H*, and Supplemental Figure S4).

**Fig. 5.**
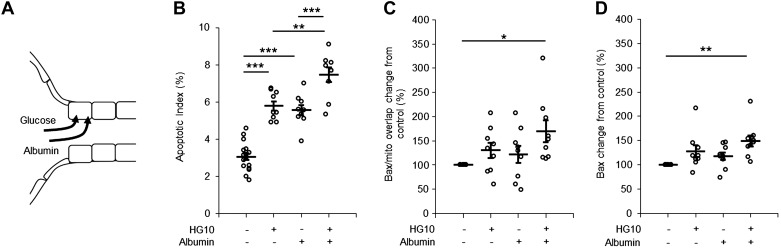
Short-time apoptotic response of proximal tubule cells (PTCs) coincubated with high glucose and albumin. *A*: cartoon illustrating the uptake of high glucose (red arrow) and albumin (purple arrow) in PTCs. *B−D*: quantification of the apoptotic index (*B*), Bax abundance (*C*), and Bax accumulation on mitochondria (*D*) in PTCs incubated with control (5.6 mM), 10 mM glucose (HG10)-, 2.5 mg/ml albumin-, or 10 mM glucose + 2.5 mg/ml albumin-containing medium for 8 h. Data are expressed as means ± SE; *n* = 9 coverslips from 3 individual cell preparations. *P < 0.05; **P < 0.01; ***P < 0.001.

Proteinuria is a hallmark of DKD. Because proteinuric kidney disease is known to be associated with PTC apoptosis (11), we next examined if coexposure to threshold concentrations of glucose and albumin would have an additive effect. Cells were exposed to glucose (10 mM) and albumin (2.5 mg/ml) either alone or in combination for 8 h (Fig. 5*A*). The albumin concentration was selected based on our previous study (11), where we showed that albumin triggers apoptosis in a dose-dependent manner in PTCs. Cells coexposed to glucose and albumin had a significantly higher AI than cells exposed to glucose or albumin alone (Fig. 5*B*). Cells coexposed to glucose and albumin, in contrast to cells exposed to either substance alone, had a significant increase in Bax abundance and Bax translocation to the mitochondria compared with control (Fig. 5, *C* and *D*).

**Fig. 6.**
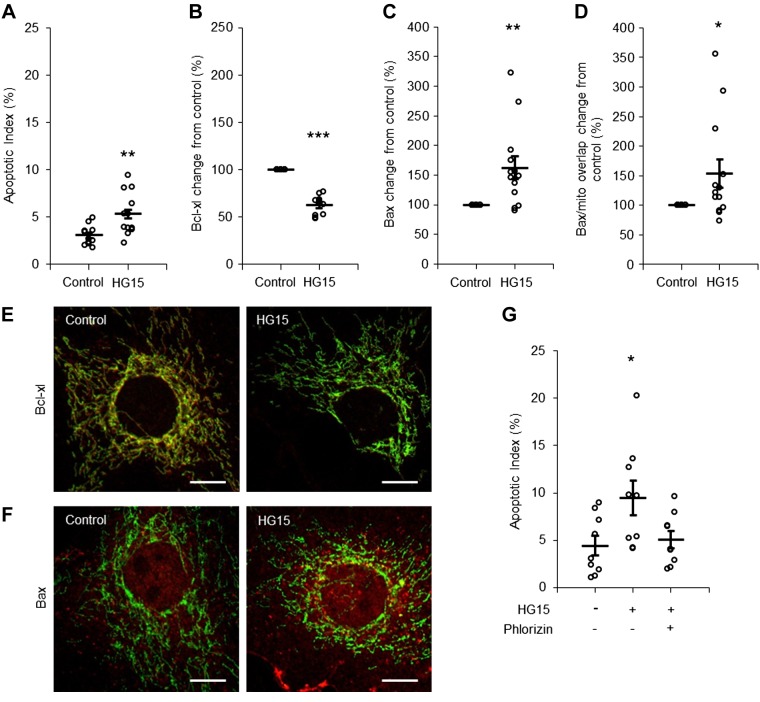
Short-time apoptotic response of mesangial cells (MCs) to an increased glucose concentration. *A−D*: quantification of the apoptotic index (AI; *A*), Bcl-x_L_ abundance (*B*), Bax abundance (*C*), and Bax accumulation on mitochondria (*D*) in MCs incubated with control (5.6 mM) or 15 mM glucose (HG15)-containing medium for 8 h. *n* = 12 coverslips from 4 individual cell preparations for the AI and Bcl-x_L_ and *n* = 13 coverslips from 5 individual cell preparations for Bax. *E* and *F*: immunofluorescence staining for Bcl-x_L_ (*E*) and Bax (*F*) expression (red) in MCs incubated with control or 15 mM glucose-containing medium for 8 h. Mitochondria are shown in green. Scale bars = 10 µm. *G*: quantification of the AI in MCs incubated with control, 15 mM glucose-, or 15 mM glucose + 0.2 mM phlorizin-containing medium for 8 h. Phlorizin was dissolved in DMSO, and an equal amount DMSO was added to all samples as a control. Data are expressed as means ± SE; *n* = 9 coverslips from 3 individual cell preparations. *P < 0.05; **P < 0.01; ***P < 0.001.

MCs also exhibited high sensitivity to short-term high glucose exposure. A significant increase in the AI was recorded in cells exposed to 15 mM glucose for 8 h (Fig. 6*A*). Activation of the mitochondrial apoptotic pathway was confirmed by decreased Bcl-x_L_ abundance, increased Bax abundance, and translocation of Bax to the mitochondria (Fig. 6, *B–F*). No apoptotic response was observed in MCs exposed to 15 mM glucose and coincubated with phlorizin, a nonselective inhibitor of SGLT1 and SGLT2 (Fig. 6*G*).

We have previously shown that subsaturating concentrations of ouabain activate an Na^+^-K^+^-ATPase/inositol 1,4,5-trisphosphate receptor signaling pathway (2) and that downstream effects involve protection from apoptosis in rat PTCs exposed to excessive concentrations of albumin (11) and Shiga toxin (10). Here, we showed that ouabain (5 nM), which has no effect on intracellular Na^+^ concentration (28), also protects from glucose-triggered apoptosis in SGLT-expressing cells (Table 1 and 2). Ouabain (5 nM) rescued from apoptosis and changes in Bax and Bcl-x_L_ expression in PTCs and MCs exposed to 15 mM glucose for 8 h and in PTCs coexposed to glucose and albumin for 8 h. Ouabain (5 nM) also protected from mitochondrial depolarization and increased ROS formation in PTCs exposed to 15 mM glucose for 4 and 8 h.

**Table 1.** Ouabain rescues PTCs and MCs from high glucose-induced apoptosis

Variable	Cell Type	Control	Glucose	Glucose + Ouabain	Number of Coverslips (Number of Preparations)
AI, %	PTC	3.1 ± 0.1	7.5 ± 0.3	2.9 ± 0.2	9 (3)
Bcl-x_L_ expression, %	PTC	100	68 ± 4	97 ± 7	15 (5)
Bax expression, %	PTC	100	155 ± 14	124 ± 9	15 (5)
Bax/mitochondrial overlap, %	PTC	100	144 ± 14	118 ± 9	15 (5)
Δψ_m_, %	PTC	100	66 ± 6	89 ± 5	3 (3)
ROS, %	PTC	100	143 ± 9	118 ± 11	8 (2)
AI, %	MC	3.1 ± 0.3	5.3 ± 0.7	3.3 ± 0.5	12 (4)
Bcl-x_L_ expression, %	MC	100	62 ± 3	97 ± 6	12 (4)
Bax expression, %	MC	100	162 ± 19	118 ± 19	13 (5)
Bax/mitochondrial overlap, %	MC	100	153 ± 24	96 ± 14	13 (5)

Values are means ± SE. Shown is the quantification of the apoptotic index (AI), Bcl-x_L_ abundance, Bax abundance and accumulation on mitochondria, mitochondrial membrane potential (Δψ_m_), and ROS in proximal tubule cells (PTCs) and mesangial cells (MCs) incubated with control (5.6 mM), 15 mM glucose-, or 15 mM glucose + 5 nM ouabain-containing medium for 8 h.

**Table 2.** Ouabain rescues PTCs from high glucose- and albumin-induced apoptosis

Variable	Cell Type	Control	Glucose + Albumin	Glucose + Albumin + Ouabain
Apoptotic index, %	PTC	3.0 ± 0.2	7.4 ± 0.4	3.4 ± 0.4
Bax expression, %	PTC	100	149 ± 12	104 ± 8
Bax/mitochondrial overlap, %	PTC	100	170 ± 22	124 ± 16

Values are means ± SE; *n* = 9 coverslips from 3 individual cell preparations. Shows is the quantification of the apoptotic index, Bax abundance, and accumulation of Bax on mitochondria in proximal tubule cells (PTCs) incubated with control (5.6 mM), 15 mM glucose + 2.5 mg/ml albumin-, or 15 mM glucose + 2.5 mg/ml albumin + 5 nM ouabain-containing medium for 8 h.

**Fig. 7.**
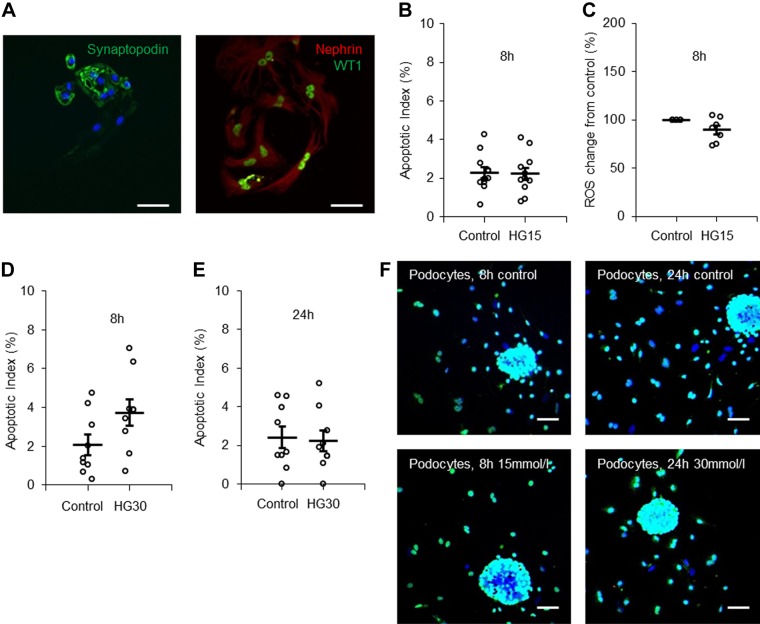
Primary podocytes do not exhibit a short-time apoptotic response to increased glucose concentration. *A*: immunostaining for podocyte-specific markers in primary podocytes. *Left*, synaptopodin (green) and DAPI (blue); *right*, nephrin (red) and Wilms tumor 1 (WT1; green). Scale bars = 10 µm. *B*: quantification of the apoptotic index in podocytes incubated with control (5.6 mM) or 15 mM glucose (HG15)-containing medium for 8 h. *n* = 12 coverslips from 4 individual cell preparations. *C*: quantification of ROS production in podocytes incubated with control or 15 mM glucose-containing medium for 8 h. *n* = 8 coverslips from 2 individual cell preparations. *D* and *E*: quantification of the apoptotic index in podocytes incubated with control or 30 mM glucose-containing medium for 8 h (*D*) or 24 h (*E*). *n* = 12 coverslips from 4 individual cell preparations. *F*: podocytes stained with TUNEL (red), WT1 (green), and DAPI (blue). Podocytes were incubated with control, 15 mM glucose, or 30 mM glucose (HG30) for 8 or 24 h as indicated. Scale bars = 40 µm. Data are expressed as means ± SE.

**Fig. 8.**
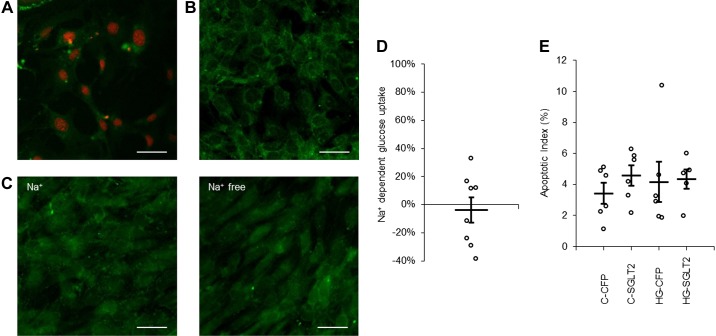
Immortalized podocytes transfected with Na^+^-dependent glucose transporter (SGLT)2 do not have Na^+^-dependent glucose uptake or increased apoptosis. *A*: immortalized podocytes transfected with SGLT2-ires-cyan fluorescent protein (CFP) (green). Nuclei were counterstained with DRAQ5 (red). Scale bar = 40 µm. *B*: immunostaining for SGLT2 (green) in immortalized podocytes transfected with SGLT2. Scale bar = 40 µm. *C*: glucose uptake in immortalized podocytes measured with 2-[*N*-(7-nitrobenz-2-oxa-1,3-diazol-4-yl)amino]-2-deoxyglucose (green) in Na^+^ or Na^+^-free buffer (5.6 mM glucose). Scale bars = 40 µm. *D*: quantification of Na^+^-dependent glucose uptake in immortalized podocytes. *n* = 8 coverslips. *E*: quantification of the apoptotic index of immortalized podocytes transfected with empty vector CFP or SGLT2 and incubated with control (C; 5.6 mM) or 15 mM glucose (HG)-containing medium for 8 h. Data are expressed as means ± SE; *n* = 6 coverslips.

Podocytes were cultured for 3 days and did at that time express the podocyte-specific proteins nephrin, synaptopodin, and WT1 (Fig. 7*A*) as well as GLUT4 (Supplemental Figure S1*C*). The culture medium contained 0.85 µM insulin. Podocytes were first exposed to 15 mM glucose for 8 h. Surprisingly, we found no increase in the AI (Fig. 7, *B* and *F*), nor was there any increase in ROS (Fig. 7*C*). The AI for podocytes was, under control conditions, in the same range as the AI for PTCs and MCs studied under control conditions (Supplemental Table S1). In the majority of previous studies on the response of immortalized podocytes to high glucose exposure, the glucose concentration has been around 25 mM or higher (25, 29, 42). Therefore, we next tested whether 8- or 24-h exposure to 30 mM glucose would provoke apoptosis in podocytes. This was not the case (Fig. 7, *D–F*). Immortalized podocytes transfected with SGLT2 vector expressed the protein (Fig. 8, *A* and *B*) but did not display Na^+^-dependent glucose uptake (Fig. 8, *C* and *D*) and did not respond with apoptosis after exposure to 15 mM glucose for 8 h (Fig. 8*E*).

## DISCUSSION

This is the first ex vivo study, to our knowledge, comparing the early response to a moderate increase in glucose concentrations in three DKD target cells. Our study highlights the importance of using primary cells for understanding the disease process and provides experimental evidence for the hypothesis that cells that are less efficient in adapting their glucose uptake are particularly vulnerable to the acute effects of hyperglycemia.

The majority of our experiments were performed on PTCs, which mainly express the high-capacity, low-affinity SGLT2 isoform. Even modest increases in extracellular glucose evoked a SGLT-dependent prompt apoptotic response in a small but significant fraction of PTCs and MCs. The apoptotic response is directly related to excessive glucose uptake via SGLT transporters, because apoptosis was not observed after inhibition or downregulation of SGLT. SGLT glucose uptake is energized by the Na^+^ gradient generated by Na^+^-K^+^-ATPase transporting three Na^+^ out of the cell and two K^+^ into the cell, at the cost of one ATP. We showed several years ago that exposure of rat proximal tubules to high glucose concentrations results in increased Na^+^-K^+^-ATPase activity and Na^+^-K^+^-ATPase-dependent energy consumption (27) (Supplemental Figure S5). This implies that Na^+^-dependent glucose transport via SGLT lacks a robust negative feedback control to protect against excessive glucose uptake. Primary podocytes had no measurable Na^+^-dependent glucose uptake and were resistant to the short-term apoptotic effects of high glucose exposure. Podocytes express GLUT4, which is located in intracellular vesicles that are translocated to the plasma membrane in an insulin-dependent manner. The insulin signaling pathway has a well-developed negative feedback control via the state of activity of several signaling proteins, including Rab-GTPase-activating. Podocytes transfected with SGLT2 vector did not exhibit Na^+^-dependent glucose uptake and did not respond to high glucose exposure with apoptosis, suggesting a more complex relationship between SGLT and Na^+^-K^+^-ATPase than what was previously believed.

The high sensitivity of PTCs to moderately increased extracellular glucose concentrations raises the question of whether PTCs are targeted already at the onset of diabetes. DKD is rarely diagnosed during the early phase of diabetes, but it is conceivable that DKD exists for a long time as an incipient disease, because the kidney has a high reserve capacity and renal epithelial cells have a relatively high regenerative capacity (17). Albuminuria is both a biomarker and risk factor in chronic kidney disease, and excessive albumin uptake in renal epithelial cells is accompanied by time- and dose-dependent activation of the mitochondrial apoptotic pathway (11). Exposure of PTCs to both albumin and glucose resulted in more extensive apoptosis than exposure to albumin or glucose alone. This finding may offer an explanation to the common clinical observation that the onset of microalbuminuria is associated with an accelerated decay of renal function (22). Because apoptosis is accompanied by increased secretion of transforming growth factor-β and other proinflammatory products that drive the fibrotic process (32, 40), we propose that acute apoptotic responses of PTCs to repeated episodes of hyperglycemia are a major cause of the progressive interstitial fibrosis in DKD. Podocytes do not regenerate and are generally considered the weak link in DKD (30, 41). Our study suggests that hyperglycemia does not exert an immediate toxic effect on podocytes and that other factors, such as exposure to glycated proteins and lack and/or resistance to insulin, are more likely to be the primary cause of podocyte injury (13, 41).

Ouabain has been demonstrated to rescue PTCs from apoptosis in animal models of proteinuric disease (11) and hemolytic uremic syndrome (10) when given in subsaturating, nontoxic concentrations. Here, we showed that ouabain (5 nM) rescued both PTCs and MCs from the onset of high glucose-triggered apoptosis. These findings have implications for the future design of a therapy that aims to halt the progressive course of DKD by preventing apoptosis.

SGLT2 inhibitors are widely used as blood glucose-lowering agents in diabetic treatment (46, 49). Their protective effect with regard to cardiovascular outcome is well documented, whereas the effect on DKD is still being evaluated (15, 35, 48, 50). Because SGLT2 inhibitors should increase the glucose load to SGLT1-expressing late PTCs, the overall renoprotective effect is difficult to predict and needs further studies. Our study points to a previously unappreciated role of SGLT transporters for diabetic complications. The vulnerability of SGLT1- and SGLT2-expressing cells to short-term exposure to increased extracellular glucose concentrations should have implications for many SGLT-expressing cell types, including cardiomyocytes, endothelial cells, and pancreatic α-islet cells (4, 5, 18). Currently, there is little information available about the acute response of these cells to high glucose exposure.

## GRANTS

This work was supported by the Swedish Research Council (to A. Aperia and H. Brismar), the Erling-Persson Family Foundation and Torsten Söderberg Foundation (to A. Aperia), and the Märta and Gunnar V. Philipsons Foundation and Magnus Bergvalls Foundation (to L. Scott). D. Svensson is supported by a Novo Nordisk postdoctoral fellowship run in partnership with Karolinska Institutet.

## DISCLOSURES

No conflicts of interest, financial or otherwise, are declared by the authors.

## AUTHOR CONTRIBUTIONS

L.M.N., L.Z., A.B., H.B., L.S., and A.A. conceived and designed research; L.M.N., L.Z., and D.S. performed experiments; L.M.N., L.Z., and D.S. analyzed data; L.M.N., L.Z., A.W., H.B., L.S., and A.A. interpreted results of experiments; L.M.N. prepared figures; L.M.N., L.S., and A.A. drafted manuscript; L.M.N., L.Z., D.S., A.W., H.B., L.S., and A.A. edited and revised manuscript; L.M.N., L.S., and A.A. approved final version of manuscript.
